# Impact of Health on Early Retirement and Post-Retirement Income Loss among Survivors of the 11 September 2001 World Trade Center Disaster

**DOI:** 10.3390/ijerph16071177

**Published:** 2019-04-02

**Authors:** Shengchao Yu, Kacie Seil, Junaid Maqsood

**Affiliations:** World Trade Center Health Registry, Division of Epidemiology, New York City Department of Health and Mental Hygiene, New York, NY 10013, USA; kseil@health.nyc.gov (K.S.); jmaqsood1@health.nyc.gov (J.M.)

**Keywords:** 9/11 impact, retirement, chronic disease, PTSD, disaster, income loss

## Abstract

The health consequences of the 9/11 World Trade Center (WTC) terrorist attacks are well documented, but few studies have assessed the disaster’s impact on employment among individuals exposed to the disaster. We examined the association between 9/11-related health conditions and early retirement among residents and workers who resided and/or worked near the WTC site on 9/11, and the association between such conditions and post-retirement income loss. The study included 6377 residents and/or area workers who completed the WTC Health Registry longitudinal health surveys in 2003–2004 and 2006–2007, and the 2017–2018 Health and Employment Survey. Logistic regression models were used to examine the associations. We found that 9/11-related health conditions were significantly associated with the likelihood of early retirement. Residents and/or area workers with more physical health conditions, especially when comorbid with posttraumatic stress disorder (PTSD), were more likely to retire before age 60 than those with no conditions. For retirees, having PTSD or PTSD comorbid with any number of physical conditions increased the odds of reporting substantial post-retirement income loss. Disaster-related outcomes can negatively impact aging individuals in the form of early retirement and income loss. Long-term effects of major disasters must continue to be studied.

## 1. Introduction

Many studies have documented the short-term health impacts of the World Trade Center (WTC) 11 September 2001 attacks, while longer-term observations of various chronic physical and mental health conditions continue to be reported among individuals exposed to the disaster [1,2,3]. However, relatively few studies have assessed the economic impact of the 9/11 attacks. It was noted that New York City (NYC) earnings at an aggregate level had $2.8 billion losses in the first three months post-9/11 [4]. For NYC industries, losses ranged from $3.6 to $6.4 billion in the nine months following the disaster [5]. Despite the well-established association between disaster exposure and impaired health among the 9/11-exposed population, few studies have examined the economic impact of 9/11-related poor health. One such study found that during the seven years after 9/11, nearly half of accidental disability retirements for NYC firefighters were for 9/11-related injuries or illnesses [6]. Another study conducted by the WTC Health Registry found a significant association between 9/11-related poor health and early retirement or job loss [7]. 

Unlike our first published work that focused on the 9/11 economic impact for non-uniformed rescue and recovery workers [7], this analysis concentrated on retirement and post-retirement well-being for residents and area workers who resided and/or worked near the WTC site on 9/11. Previous research showed that different groups of survivors, such as rescue and recovery workers, local residents, area workers, passersby, and students and school staff, may have had different health outcomes, both physical and mental, due to their varying levels of disaster exposure [1,8,9]. Consequently, health-related retirement may also differ for different groups of survivors. Furthermore, retirement options available to rescue and recovery workers, especially uniformed workers, as compared to residents and area workers, can be a key factor in making retirement decisions and must be linked to the subsequent economic outcome [10,11,12,13]. 

Retirement trends were shifting during the mid-1990s into the 2000s irrespective of the effects of the 9/11 disaster, as fewer people were retiring at younger ages compared to the 1970s and 1980s [14]. Trends of employer pension plans becoming rarer, a healthier population overall, fewer labor-intensive jobs, less employer-sponsored health benefits for retirees under age 65, and an older full retirement age for Social Security have resulted in people retiring at older ages than earlier generations [15]. In tandem, retirement patterns have become more complex with many individuals transitioning from full-time to part-time work or working again after retirement [15]. Early retirement, therefore, is an important factor to consider for economic assessments, as it can strongly affect one’s well-being (such as post-retirement income) for the remainder of one’s life. 

Poor health has been linked to early retirement in numerous studies for different population groups or countries. Self-perceived poor health was a risk factor for early exit from the labor force through early retirement, unemployment, or both [16,17,18,19,20]. Other studies found an association between a variety of health conditions, such as respiratory, cardiovascular, musculoskeletal diseases, cancer, and other chronic conditions, and premature labor force exit [16,17,19,21,22,23]. However, health problems are not the only factor that impacts decisions about early retirement; one’s financial situation is another key factor in this decision-making process [24,25]. Experiencing a layoff or having a spouse who retires early increases one’s likelihood of retiring early whereas being able to take a new job that requires fewer hours or offers higher pay is protective against retiring early [26]. Multiple studies have concluded, however, that health, even a subjective assessment of health, is the most important predictor of early retirement [24,25,26]. 

Previous studies of 9/11 health impact showed elevated prevalence of many chronic physical conditions, posttraumatic stress disorder (PTSD), and physical-mental comorbidity among disaster survivors [1,9,27,28,29,30,31,32]. We examined 9/11-related health conditions and PTSD comorbidity in the current analysis of health’s impact on early retirement. We also went one step further and assessed whether or not those physically and/or mentally affected by the disaster continued to experience decline in their well-being after retirement in terms of substantial income loss.

In 2017–2018, the WTC Health Registry conducted an in-depth study on health and employment for a sub-sample of its enrollees. Detailed information on employment, retirement, health insurance, and other economic indicators collected from this survey, coupled with detailed health data collected from the Registry’s longitudinal surveys administered in waves since 2003, provided an opportunity to further investigate the impact of 9/11-related health on retirement and post-retirement well-being. As in our previous study [7], the number of 9/11-related chronic physical health conditions and PTSD were used to measure post-9/11 health status. We hypothesized that the more 9/11-related health conditions an individual had, the more likely he or she would be to retire before age 60. Furthermore, for those who had already retired, the number of post-9/11 conditions they suffered was associated with the degree of their post-retirement income loss.

## 2. Materials and Methods

### 2.1. Study Population

The WTC Health Registry maintains a longitudinal cohort that was established in 2002 following the 11 September 2001 terrorist attacks with the aim of monitoring the long-term health outcomes in individuals exposed to the events of 9/11 in NYC. In 2003–2004, the Registry enrolled 71,426 rescue or recovery workers, Lower Manhattan residents, area workers, passersby, and students or school staff by conducting its first health survey (Wave 1). Eligible enrollees have since been invited to participate in three additional health surveys in 2006–2007 (Wave 2), 2011–2012 (Wave 3), and 2015–2016 (Wave 4) as well as a number of in-depth studies. Previous Registry publications provide a more detailed description of this cohort [1,8]. 

From September 2017 to March 2018, the Registry conducted the Health and Employment Survey (HES) with a sample pool of English-speaking enrollees under 75 years of age (as of 2017) who completed at least Waves 1 and 2. Enrollees who reported retirement or unemployment due to disability/health at any of the three follow-up surveys (Waves 2–4) were invited to participate in the HES study along with a roughly equivalent number of not-yet retired age-matched enrollees. In total, 23,036 enrollees were invited to complete the HES, and the response rate reached 65%. The US Centers for Disease Control and Prevention (3793) and the NYC Department of Health and Mental Hygiene (02058) institutional review boards approved the Registry protocol and the HES protocol (17047), including the use of the data. 

This study used data collected from the HES and was limited to those who lived or worked near the WTC site on 11 September 2001. During analyses, the HES data were merged with the Registry’s four waves of health survey data to obtain 9/11-related health information for the study sample. We excluded 341 enrollees who had retired before the 9/11 disaster, as our focus was on the link between 9/11-related health and retirement. The final study sample was 6377 enrollees, which included 3486 retired and 2891 not-yet-retired enrollees. Of the 3486 retirees, 1234 retired before age 60, 2143 retired at or after age 60, and 109 did not report time of retirement. Additionally, 2395 enrollees reported post-retirement income loss.

### 2.2. Study Outcomes: Early Retirement and Post-Retirement Income Loss

In the HES, enrollees were asked if they were currently retired and, if retired, for the month and year of retirement. This information was used to calculate retirement age. In the analytical model, a dichotomous outcome variable was created for early retirement which was defined as having retired before reaching the age of 60. 

Those self-reporting retirement in the HES were also asked if their total post-retirement personal income after taxes changed as compared to pre-retirement and in which direction; if they responded that their income had decreased, a follow-up question asked for the percentage of decrease (<25%, 25% to 50%, >50%). A dichotomous outcome variable for post-retirement income loss was created for those who reported substantial income loss which was defined as having over 50% of income decrease. 

### 2.3. Chronic Physical Health Conditions and PTSD Measure

Starting in Wave 1, Registry enrollees were asked in each of the follow-up wave surveys whether they had ever been told by a doctor or other health professional that they had any of more than a dozen listed health conditions and the year of first diagnosis if diagnosed. For this study, asthma, heart disease (coronary heart disease, angina, heart attack, or other heart disease), stroke, lung disease (emphysema, chronic bronchitis, reactive airways dysfunction syndrome, sarcoidosis, pulmonary fibrosis, or other lung disease), diabetes, gastroesophageal reflux disease, and autoimmune disease (multiple sclerosis or amyotrophic lateral sclerosis, rheumatoid arthritis, or other autoimmune disease) were selected, as all have been reported to be elevated among 9/11 exposed individuals [27,33,34,35,36,37,38,39]. If an enrollee reported a diagnosis for any of the seven selected types of conditions in or after 2001, we categorized them as having a 9/11-related chronic physical health condition. Probable PTSD was assessed at every wave of the Registry survey via a 9/11-specific PTSD Checklist-Civilian Version (PCL-17) scale. A cut-off score of 44 or greater at any wave was used to define probable PTSD as in prior published Registry studies [1]. Co-occurrence of any of the seven selected types of physical conditions and probable PTSD was then defined as 9/11-related physical–mental comorbidity in this study. 

The total number of chronic physical conditions, comorbid with PTSD or not, was used as the health indicator in the analytical models.

### 2.4. Sociodemographic Characteristics and Other Covariate Measures

Sociodemographic characteristics of the study sample were measured by sex, age at 11 September 2001, race/ethnicity, household income, education, and marital status. A quantitative 9/11 exposure measure was also included in the analysis as it is closely associated with PTSD [1]. The exposure measure combines disaster information, such as dust cloud exposure, injury, witnessing horror, bereavement, and home evacuation experience, collected from both Waves 1 and 2 into a 12-item score that was collapsed into four categories: 0–1 as no/low exposure, 2–3 as medium, 4–5 as high, and six or greater as very high exposure.

### 2.5. Statistical Analyses

Two logistic regression models were used to calculate the adjusted odds ratio (AOR) and 95% confidence intervals (95% CI) to measure the association between 9/11-related health conditions and our study outcomes. The equation for these models is

(1) $$\mathrm{logit}\left(p\right)=\ln\left(\frac{p}{1-p}\right)=\beta_{0}+\beta_{1}X_{1}+\beta_{2}X_{2}+\cdots +\beta_{k}X_{k}$$

where *p* is the probability of our outcome occurring, $\beta_{0}$ is the intercept, $\beta_{1}$, …, $\beta_{k}$ are the regression coefficients, and $X_{1}$, …, $X_{k}$ are the predictors or covariates included in the model (described above). The adjusted odds ratio was estimated by taking the exponential of the regression coefficients in the model.

The first model included 4125 non-retired enrollees and early retirees, and aimed to demonstrate the relationship between poor health (measured by the number of 9/11-related health conditions with and without PTSD) and the likelihood of retiring before age 60, while adjusting for sociodemographic factors and disaster exposure.

The second model included 2395 enrollees who reported income loss after retirement and investigated the association between 9/11-related health conditions and substantial post-retirement income loss while adjusting for disaster exposure and all sociodemographic characteristics included in the first model except for age at 11 September 2001. Even though post-retirement income decreases are common and subject to lower tax rates and less work-related expenses [40], this analysis aimed to reveal that substantial income decrease (percentage of decrease larger than 50%) was associated with health.

The analyses for this study were conducted in SAS version 9.4 (SAS Institute Inc., Cary, NC, USA).

## 3. Results

Among 4125 residents and area workers who lived or worked near the WTC site on 9/11, 1234 (29.9%) reported having retired before age 60 (Table 1). Sociodemographic characteristics that were significantly associated with early retirement included age, race/ethnicity, total household income, marital status, and education. Not surprisingly, younger enrollees were less likely to report that they had retired under age 60, as they may have not yet retired at all. Compared to non-Hispanic white residents and area workers, non-Hispanic black and Hispanic residents and area workers both had higher odds of reporting early retirement (AOR = 1.7, 95% CI: 1.3–2.1; AOR = 1.3, 95% CI: 1.0–1.7). Enrollees who were in the three higher household income groups ($50,000–<$75,000, $75,000–<$150,000, and ≥$150,000) each had higher likelihoods of experiencing early retirement. Those in the highest income group (≥$150,000) were more than twice as likely as those in the lowest income group (<$25,000) to have early retirement (AOR = 2.2, 95% CI: 1.5–3.2). On the contrary, the likelihood of reporting early retirement dropped by about 40% (AOR = 0.6, 95% CI: 0.4–0.7) and 70% (AOR = 0.3, 95% CI: 0.3–0.4), respectively, for those with a college or post-graduate degree compared to those with high school and below education. Enrollees who were widowed had a higher likelihood of experiencing early retirement compared to those who were married or living with a partner (AOR = 1.5, 95% CI: 1.0–2.3). Sex and 9/11-related disaster exposure did not show significant association with early retirement for residents and area workers.

Having chronic 9/11-related physical health conditions without PTSD was found to be significantly associated with early retirement in a dose-response manner with the odds of reporting early retirement increasing as the number of conditions increased (AOR = 1.5, 95% CI: 1.2–1.8; AOR = 1.4, 95% CI: 1.1–1.9; AOR = 1.9, 95% CI: 1.2–3.0 for those with one, two, and three or more conditions without PTSD, respectively) (Table 1). When chronic physical conditions were comorbid with PTSD, the likelihood of experiencing early retirement increased further and also in a dose-response manner. For example, for residents or area workers who had three or more 9/11-related physical conditions and PTSD, their odds of reporting early retirement were more than three times higher than those who did not have any of the selected conditions or PTSD (AOR = 3.4, 95% CI: 2.4–4.7). Interestingly, having PTSD alone did not significantly increase one’s odds of early retirement as compared to those who did not have any physical conditions (AOR = 1.2, 95% CI: 0.9–1.6). To test the robustness of the association of 9/11-related health and early retirement, we performed a sensitivity analysis using an Ordinary Least Squares model by replacing the dichotomous early retirement outcome variable with a continuous variable measuring actual age at retirement. Our results showed findings consistent with the early retirement model results: The coefficients for enrollees who suffered one, two, or three or more physical conditions comorbid with PTSD (coefficients = −1.82, −2.89, and −2.92, respectively, and all with *p* < 0.0001) were all significant with the retirement age decreasing by an average of two to three years compared to those who did not have any 9/11-related chronic conditions (results not shown).

Approximately 73% of retirees in the study sample reported that their post-retirement income had decreased as compared to their pre-retirement income, while 19% reported no significant income changes, and 8% reported increased post-retirement income. Although post-retirement income loss seemed to be a norm for retirees regardless of their retirement age, among those who reported decreased income, a significantly higher proportion (45.9% vs. 33.7% in Table 2) of early retirees reported an income decrease of more than 50% (*p* < 0.0001). Because of the significant association of 9/11-related poor health and early retirement demonstrated in Table 1, we further tested if 9/11-related poor health was directly linked to retirees’ post-retirement financial status. 

Table 3 presents results on the association between 9/11-related health and the likelihood of having more than 50% of income decrease after retirement among enrollees who reported decreased income. Only three sociodemographic characteristics were associated with substantial post-retirement income loss: race/ethnicity, total household income, and education. More specifically, non-Hispanic black residents and area workers were 30% less likely to report income loss greater than 50% as compared to non-Hispanic white residents and area workers (AOR = 0.7, 95% CI: 0.6–1.0). Other racial/ethnic groups did not show significantly higher or lower odds of experiencing substantial income loss compared to non-Hispanic whites. Compared to the lowest income group (<$25,000), the three middle income groups ($25,000–<$50,000, $50,000–<$75,000, and $75,000–<$150,000) were less likely to have substantial income loss (AOR = 0.5, 95% CI: 0.3–0.8; AOR = 0.6, 95% CI: 0.3–0.9; AOR = 0.6, 95% CI: 0.4–1.0, respectively); however, the highest income group (≥$150,000) did not show significantly higher or lower odds of experiencing substantial income loss. Enrollees with some college experience or a college degree had a significantly higher likelihood of substantial income loss compared to enrollees with lower education attainment (AOR = 1.2, 95% CI: 1.0–1.6). Each level of 9/11-related disaster exposure was highly associated with post-retirement income loss in a dose-response manner. The highest level of exposure increased the odds of having substantial income loss by 2.5 times compared to those with little or no exposure (AOR = 2.5, 95% CI: 1.7–3.6).

Substantial post-retirement income loss was also significantly associated with the number of 9/11-related health conditions but only for those who suffered from PTSD alone or PTSD comorbid with physical conditions. In other words, enrollees without PTSD who only had chronic physical health conditions did not have higher odds of losing more than 50% of their income post-retirement compared to those who had no health conditions. Once enrollees had PTSD, their likelihood of having substantial income loss increased significantly as compared to those without any health conditions (e.g., AOR = 1.7, 95% CI: 1.2–2.5). PTSD comorbid with one or more physical health conditions had a similar effect on enrollees’ odds of experiencing substantial income loss as PTSD alone.

## 4. Discussion

The impact of the 9/11 disaster on early retirement and post-retirement well-being among survivors has rarely been studied until now. This study examined the association of 9/11-related health and early retirement for residents and area workers who resided and/or worked near the WTC site on 9/11. Furthermore, we assessed the longer-term impact of 9/11-related health on post-retirement income loss among survivors.

Sixteen years after the 9/11 disaster, among the 3486 retired residents and area workers in our study sample, about 35% had retired before reaching 60 years of age. Early retirement was found to be significantly associated with chronic 9/11-related physical health conditions in a dose-response manner, and a sensitivity analysis estimating the magnitudes of the effects in terms of years of work lost found consistent results. More specifically, the likelihood of reporting early retirement versus not-yet retired grew as the number of physical health conditions increased. The odds of reporting early retirement increased further when these conditions were comorbid with PTSD but not when PTSD was reported in the absence of any 9/11-related physical conditions. These findings suggest that residents or area workers who suffered from 9/11-related PTSD but not other chronic physical conditions were not more likely to retire early due to this mental health condition alone; several explanations may clarify this. One reason is that PTSD may not severely impact daily working life, especially if the PTSD is mild. Only 6% of the early retirees in this study sample reported having Accidental Disability Retirement (ADR), a benefit available to members of certain plans if they become permanently physically or mentally incapacitated and are unable to perform the duties of their job due to an accident that took place on the job [41]. The percentage of early retirees with ADR and PTSD but no other chronic condition is even lower (4%), which suggests the proportion of enrollees with severe PTSD alone is low. Another potential reason is that stigma associated with a mental health condition such as PTSD may prevent one from recognizing the condition, let alone coping with it by retiring early.

Despite the fact that PTSD alone did not show a significant association with reporting early retirement, this study found that residents and area workers with PTSD alone or PTSD comorbid with any number of physical conditions were significantly more likely to experience substantial income loss post-retirement. PTSD comorbid with one or more physical health conditions had a similar effect, in terms of significance and magnitude, on the odds of experiencing substantial income loss as PTSD alone. Having any number of physical conditions (including three or more conditions) in the absence of PTSD was not significantly associated with substantial post-retirement income loss, which suggests PTSD is the key and driving factor in this association. The economic impact of 9/11-related PTSD, although not directly reflected in the form of early retirement, tended to be stronger in the long-term as individuals suffering PTSD were less likely to assume income-generating activities that could ultimately lead to significant income loss. Like having PTSD alone, disaster exposure did not show a significant association with early retirement but was highly related to post-retirement income loss. The absence of exposure’s effect in the model for early retirement is likely a result of having the 9/11-related health measure in the same model absorbing its effect. We re-ran our model for Table 1 without the health measure and found that disaster exposure at a very high level was significantly associated with 50% higher likelihood of early retirement (AOR = 1.5, 95% CI: 1.1–2.0, results not shown) compared to those who had low or no exposure during the disaster. This finding implies that the health measure we chose for this study represented the impact of this disaster well. A closer look at the data suggested that enrollees in the highest category of the 9/11 exposure scale were most likely to have suffered PTSD or PTSD comorbid with physical health conditions (results not shown) which made their comparable associations with the two outcomes unsurprising. 

Similar to the findings in our earlier study [7], those with higher household income or lower education were more likely to report early retirement than staying in the labor force as compared to the low income group or higher education achievers. This seemingly contradictory impact of high income and low education reflects the complexity of the retirement decision-making process. For residents and area workers with 9/11-related poor health, having a higher income may have allowed them to voluntarily retire early or made them more likely to afford early retirement without considerable additional financial concern compared to having a lower income. On the other hand, the early retirement decision for the lower education group was less likely to be a “choice”, as their low education (usually accompanied with low income) and poor health may not have allowed them to maintain their work, especially when the work was more physical or manual in nature. The association of lower socioeconomic status and higher risk of involuntary labor force exit was also found in previous studies [42,43]. 

This study provided evidence of a strong association between 9/11-related poor health and early retirement among survivors who lived and worked near the WTC site. Furthermore, with additional information collected from the HES, we demonstrated, for the first time, the significant impact of 9/11-related PTSD and 9/11-related physical–mental comorbidity on substantial post-retirement income loss. Our findings suggest that after labor force exit, poor health, especially PTSD, continued to adversely affect survivors’ overall well-being in the form of considerable income reduction. The compromised post-retirement well-being as a result of poor health did not come as a surprise because many of the affected people could have involuntarily experienced early retirement before securing their full retirement benefits; or, they might be less likely to participate in any income-generating activities, particularly if they experienced mental health or physical–mental comorbidity issues after retirement. This lasting effect of poor health, along with the fact that certain race/ethnicity (Hispanic and non-Hispanic black) and low education groups are more likely to retire early, should direct future 9/11-related resources by placing a greater focus on limiting sharp income loss and providing adequate and affordable health care services to retirees from various vulnerable groups to maintain a certain level of quality of life. 

One notable advantage of this study is that we directly assessed the relationship between 9/11-related health and post-retirement income loss using statistical modeling. This investigation provides the first look at one aspect of survivors’ well-being beyond retirement and will lead to additional exploration of the economic impact of 9/11, such as the relationship between substantial income loss and worsening health and its associated high medical cost, poor health reducing survivors’ ability to re-enter the workforce to compensate for post-retirement income loss, and how survivors’ retirement plans directly affect their post-retirement well-being. In addition, future research should also assess other aspects of post-retirement well-being such as retirees’ current health status or health-related quality of life (as in an ongoing study by our group) and insurance coverage. These results will not only give us a broader picture of retirees’ overall well-being but also help estimate the realistic gap on health care access in terms of making policy recommendations.

## 5. Conclusions

This study found that 9/11-related health conditions were significantly associated with the likelihood of early retirement among residents and area workers who survived the 9/11 attacks. The likelihood of reporting early retirement increased as the number of health conditions increased. Having PTSD alone was not associated with early retirement; however, PTSD was the driving factor out of all 9/11-related health conditions studied that was linked to substantial income loss post-retirement. For future studies of 9/11 economic impact, our findings emphasize the key role that PTSD may play in the longer-term.

## Figures and Tables

**Table 1:** Early retirement, chronic physical health conditions and post-traumatic stress disorder (PTSD) comorbidity, and other characteristics among non-retired and early retired enrollees.

Sample Characteristics	Retirement Status				Likelihood of Early Retirement	
	Not Retired (*N*, %)		Retired Early (*N*, %)			
	2891	70.1	1234	29.9	AOR ^a^	95% CI ^b^
Sex						
Female	1481	68.1	693	31.9	Ref	
Male	1410	72.3	541	27.7	1.0	0.8, 1.1
Age on 9/11						
0–24	31	93.9	2	6.1	0.1	0.0, 0.6
25–44	1089	79.1	287	20.9	0.5	0.4, 0.6
45–64	1771	65.2	945	34.8	Ref	
Race/ethnicity						
Non-Hispanic white	2085	72.1	805	27.9	Ref	
Non-Hispanic black	304	59.3	209	40.7	1.7	1.3, 2.1
Hispanic	253	64.4	140	35.6	1.3	1.0, 1.7
Non-Hispanic Asian	155	77.9	44	22.1	0.9	0.6, 1.3
Other race or multi-racial	94	72.3	36	27.7	0.9	0.6, 1.4
Total household income in 2002, $						
<25,000	167	72.3	64	27.7	Ref	
25,000 to <50,000	473	70.0	203	30.0	1.1	0.8, 1.6
50,000 to <75,000	477	68.3	221	31.7	1.6	1.1, 2.3
75,000 to <150,000	924	71.2	373	28.8	1.6	1.1, 2.3
≥150,000	564	71.4	226	28.6	2.2	1.5, 3.2
Education						
High school diploma or lower	335	54.1	284	45.9	Ref	
Some college or college graduate	1583	70.0	677	30.0	0.6	0.4, 0.7
Post-graduate	964	78.2	268	21.8	0.3	0.3, 0.4
Marital status						
Married or living with partner	1852	69.8	803	30.2	Ref	
Divorced or separated	415	69.5	182	30.5	0.9	0.7, 1.1
Widowed	57	53.8	49	46.2	1.5	1.0, 2.3
Never married	552	74.1	193	25.9	0.9	0.7, 1.2
Disaster exposure						
Low/none	630	70.6	262	29.4	Ref	
Medium	1264	70.9	520	29.1	0.9	0.8, 1.1
High	784	70.8	323	29.2	0.8	0.7, 1.1
Very high	213	62.3	129	37.7	1.1	0.8, 1.5
Number of chronic conditions ^c^ without PTSD						
0	1289	76.8	390	23.2	Ref	
1	527	68.7	240	31.3	1.5	1.2, 1.8
2	193	67.2	94	32.8	1.4	1.1, 1.9
≥3	61	59.8	41	40.2	1.9	1.2, 3.0
Number of chronic conditions ^c^ with PTSD						
0	324	73.6	116	26.4	1.2	0.9, 1.6
1	238	65.2	127	34.8	1.7	1.3, 2.2
2	153	57.1	115	42.9	2.5	1.9, 3.4
≥3	106	48.8	111	51.2	3.4	2.4, 4.7

^a^ AOR: Adjusted odds ratio and was adjusted for all factors listed in this table. ^b^ 95% CI: 95% confidence interval. ^c^ Chronic physical health conditions include asthma, heart diseases, stroke, lung diseases, diabetes, gastroesophageal reflux disease (GERD), and autoimmune diseases.

**Table 2:** Percentage of post-retirement income loss among retirees reporting decreased income.

Retirement Age (Years)	Post-Retirement Income Loss, %		Total, *N*
	≤50% (*N* = 1437)	>50% (*N* = 889)	2326
<60	54.1	45.9	859
≥60	66.3	33.7	1467

**Table 3:** Post-retirement income loss, chronic physical health conditions and post-traumatic stress disorder (PTSD) comorbidity, and other characteristics among retirees reporting decreased income.

Sample Characteristics	Post-Retirement Income Loss more than 50%				Likelihood of Substantial Income Loss	
	No (*N*, %)		Yes (*N*, %)			
	1469	61.3	926	38.7	AOR ^a^	95% CI ^b^
Sex						
Female	787	63.3	456	36.7	Ref	
Male	682	59.2	470	40.8	1.0	0.8, 1.2
Race/ethnicity						
Non-Hispanic white	990	59.1	685	40.9	Ref	
Non-Hispanic black	234	69.6	102	30.4	0.7	0.6, 1.0
Hispanic	145	66.5	73	33.5	0.8	0.5, 1.1
Non-Hispanic Asian	57	58.8	40	41.2	1.1	0.7, 1.7
Other race or multi-racial	43	62.3	26	37.7	1.0	0.5, 1.7
Total household income in 2002, $						
<25,000	46	47.9	50	52.1	Ref	
25,000 to <50,000	255	68.5	117	31.5	0.5	0.3, 0.8
50,000 to <75,000	280	67.3	136	32.7	0.6	0.3, 0.9
75,000 to <150,000	499	64.4	276	35.6	0.6	0.4, 1.0
≥150,000	226	48.3	242	51.7	1.3	0.8, 2.1
Education						
High school diploma or lower	299	65.9	155	34.1	Ref	
Some college or college graduate	780	60.0	521	40.0	1.2	1.0, 1.6
Post-graduate	380	60.8	245	39.2	1.0	0.8, 1.4
Marital status						
Married or living with partner	961	60.7	623	39.3	Ref	
Divorced or separated	236	59.9	158	40.1	1.2	0.9, 1.6
Widowed	66	71.0	27	29.0	0.7	0.4, 1.1
Never married	195	63.1	114	36.9	1.0	0.8, 1.4
Disaster exposure						
Low/none	362	71.7	143	28.3	Ref	
Medium	653	63.6	373	36.4	1.4	1.0, 1.7
High	360	55.8	285	44.2	1.7	1.2, 2.2
Very high	94	42.9	125	57.1	2.5	1.7, 3.6
Number of chronic conditions ^c^ without PTSD						
0	546	65.7	285	34.3	Ref	
1	316	64.6	173	35.4	1.1	0.9, 1.5
2	129	68.6	59	31.4	0.9	0.6, 1.3
≥3	59	62.8	35	37.2	1.4	0.9, 2.2
Number of chronic conditions ^c^ with PTSD						
0	118	53.2	104	46.8	1.7	1.2, 2.5
1	114	53.0	101	47.0	1.8	1.2, 2.5
2	104	55.9	82	44.1	1.6	1.1, 2.3
≥3	83	48.8	87	51.2	1.9	1.3, 2.7

^a^ AOR: Adjusted odds ratio and was adjusted for all factors listed in this table. ^b^ 95% CI: 95% confidence interval. ^c^ Chronic physical health conditions include asthma, heart diseases, stroke, lung diseases, diabetes, gastroesophageal reflux disease (GERD), and autoimmune diseases.
